# Emerging personalized virtual brain models: next-generation resection neurosurgery for drug-resistant epilepsy?

**DOI:** 10.1186/s42494-023-00128-1

**Published:** 2023-07-07

**Authors:** Qiao Wang, Guangyuan Jin, Tao Yu, Fabrice Bartolomei, Liankun Ren

**Affiliations:** 1 Department of Neurology, Xuanwu Hospital, Clinical Center for Epilepsy, Capital Medical University, Beijing, 100053 China; 2 National Center for Neurological Disorders, Beijing, 100070 China; 3 Department of Functional Neurosurgery, Beijing Institute of Functional Neurosurgery, Xuanwu Hospital, Clinical Center for Epilepsy, Capital Medical University, Beijing, 100053 China; 4 APHM, Epileptology and Clinical Neurophysiology Department, Timone Hospital, Marseille, 13005 France; 5 Aix-Marseille Université, Institut National de la Santé et de la Recherche Médicale, Institut de Neurosciences des Systèmes (INS) UMR1106, Marseille, 13005 France; 6 Chinese Institute for Brain Research, Beijing, 102206 China; 7 Department of Neurology, Xuanwu Hospital, Capital Medical University, NO.45 Changchun Street, Xicheng District, Beijing, 100053 China

**Keywords:** Drug-resistant focal epilepsy, Personalized virtual brain models, Machine learning, Epileptogenic zone networks, Stereoelectroencephalography, Virtual surgery

## Abstract

Recently, a novel workflow known as the virtual epileptic patient (VEP) has been proposed by a research team from Aix Marseille Université in their papers published in *Lancet Neurology*, *Science Translational Medicine* and *Epilepsia*. This method involves creating an individualized virtual brain model based on computational modelling, which can facilitate clinical decision-making by estimating the epileptogenic zone and performing the virtual surgery. Here, we summarize brief workflow, strengths, and limitations of VEP, as well as its performance in a retrospective study of 53 patients with drug-resistant focal epilepsy who underwent stereoelectroencephalography. A large-scale clinical trial (NCT03643016) is underway to further assess VEP, which is expected to enroll 356 patients prospectively. As supporting evidence accumulates, the clinical application of VEP has the potential to improve clinical practice, leading to better outcomes and qualities of life of patients.

## Background

Epilepsy is a common neurological disorder characterized by recurring seizures that can significantly impact a person’s quality of life [1]. Approximately 30% of patients suffer from drug-resistant focal epilepsy, making them candidates for surgical treatment [2]. Although enormous efforts have been made to improve postsurgical outcomes over half a century, including technical advances in multimodality neuroimaging and intracranial recordings, only a modest increase of seizure freedom after surgery has been achieved [3]. In the current practice, epileptogenic zone networks (EZNs) are localized mainly by integrated information from seizure semiology, neuroimaging and electrophysiology, which are individually weighted and assessed mainly based on clinical experience. Artificial intelligence and computational sciences are, therefore, anticipated to inform localization of EZNs and precisely predict surgical outcome.

## Main text

Recently, a research team from Aix Marseille Université proposed a novel workflow named the virtual epileptic patient (VEP), in their papers published in *Lancet Neurology*, *Science Translational Medicine* and *Epilepsia* [4–6]. With this new workflow, they constructed a personalized virtual brain model using computational modelling methods to aid in localization of EZNs for surgical intervention and predicting surgical outcomes. Unlike the traditional model-free EZN quantification methods based on spectral analysis of stereoelectroencephalography (SEEG) signals such as the epileptogenicity index (EI) [7] and subsequent connectivity EI (cEI) [8], this model-based approach can simulate brain functional data at any site within the entire cerebral space, thereby overcoming the sparsity of spatial sampling in various types of intracranial recordings. Besides, this personalized virtual brain can be further used to test therapeutic effects of brain stimulation and virtual surgery by simulating different scenarios and interventions.

**Fig. 1:**
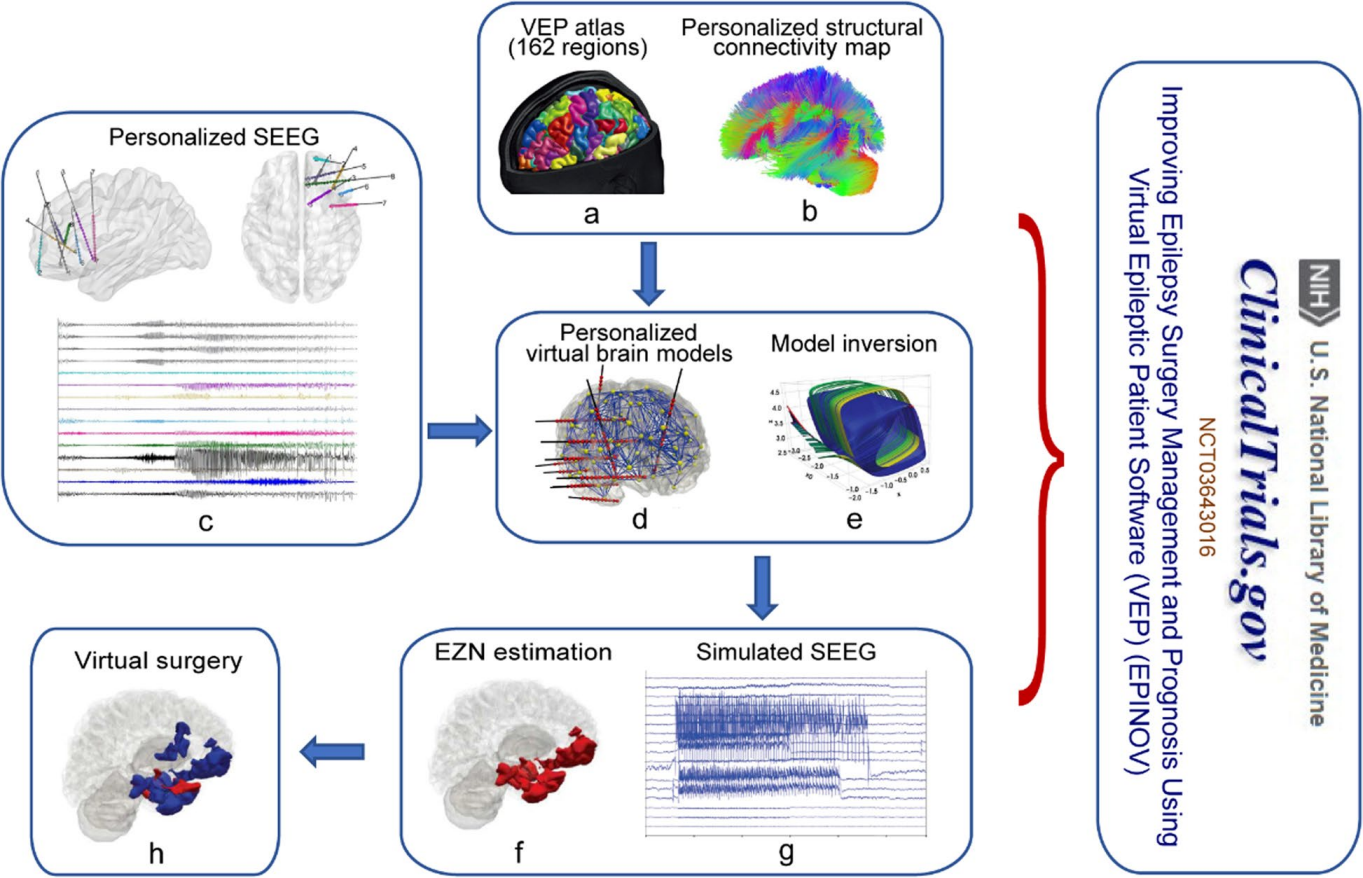
Application of personalized virtue brain modeling in drug-resistant epilepsy: from bench to bedside. First, a T1-weighted MRI is utilized to acquire brain anatomy and delineate distinct brain regions based on the Virtual Epileptic Patient atlas (**a**) as the nodes in the network model. The links between the nodes of the network are estimated based on patient-specific structural connectivity map (**b**) calculated from a diffusion-weighted imaging. Then, each node was assigned a neural mass model to simulate the average neuronal activity at that node. The Bayesian inference methods are used to estimate the patient-specific parameters of each NMM by fitting the simulated source activity (**g**) to the corresponding SEEG signals (**c**) with consideration of prior knowledge, a process called model inversion (**e**). Finally, a personalized brain model is constructed (**d**) and the output of the VEP workflow is the suggested epileptogenic zone networks (**f**), and the personalized model can be used to test different surgical strategies (**h**). Permission was granted by Viktor Jirsa et al. (©Elsevier [5]) to reuse this figure (**a**, **d** and **g**). Permission was granted by Huifang E. Wang et al. (©American Association for the Advancement of Science [4]) to reuse this figure (**e**, **f** and **h**)

The workflow of VEP is as follows (Fig. 1). First, the personalized virtual brain network model is constructed based on individual anatomical data from magnetic resonance imaging (MRI) (T1 and diffusion-weighted imaging, DWI). Then, the brain is defined as a network of regions delineated by a brain atlas (e.g., the VEP atlas), and each region is represented as a node in the network model. The VEP atlas is a modified cortical and subcortical parcellation of the brain based on the Freesurfer Destrieux atlas that considers region sizes adapted to EZN diagnostics [9]. The computational model, a so-called neural mass model (NMM), is used to calculate the average neuronal activity at each node. Both the connectivity between nodes and the parameter settings of the NMM assigned to each node (e.g., excitability) play crucial roles in generating seizure activity. The links between the nodes of the network are estimated based on patient-specific structural connectivity matrix calculated from DWI. Then, the Epileptor model, a non-linear mathematical model developed based on seizure dynamics and can mimic the predominant seizure class, is used to simulate the seizure-like activities [8]. By fitting the signals generated by the Epileptor model to the recorded signals from SEEG, the Bayesian inference methods are used to estimate the patient-specific parameters of each NMM, with consideration of the data features extracted from SEEG signals and prior knowledge, such as MRI-identifiable lesions or the clinically defined EZNs. Finally, a personalized brain model is constructed using individual structural and functional information. The model is further used to estimate the EZN and test different surgical strategies.

In a recent retrospective study, the performance of VEP was evaluated in 53 patients with drug-resistant focal epilepsy who had undergone SEEG. The results demonstrated a precision rate of 64% in identifying regions as epileptogenic compared to clinical analysis [6]. Moreover, the EZNs identified by VEP showed a small physical distance from clinically defined EZNs. VEP exhibited a higher precision rate in patients with a seizure-free outcome after surgical resection than in the non-seizure-free patients. Additionally, the regions identified as epileptogenic by VEP might include regions not sampled by SEEG electrodes. The non-resected VEP epileptogenic regions were more numerous in the non-seizure-free patients in comparison with the seizure-free patients. VEP is now undergoing further evaluation in a large-scale clinical trial (EPINOV, NCT03643016) with an expected enrollment of 356 prospective patients with drug-resistant focal epilepsy [5].

Mathematical modelling and computational tools are increasingly applied in clinical practice, such as for diagnosing neurological disorders and predicting the prognosis. These techniques have main advantages of the reproducibility and objectivity of the results. VEP has the potential to improve clinical decision-making, particularly with respect to accurate localization of EZNs and surgical planning. However, it is important to note that there are some limitations and challenges associated with VEP in the current stage [4]. One of the main challenges in translating VEP to clinical use is the long computation time required for its implementation. Furthermore, the utilization of VEP demands a high level of expertise, which presents another obstacle to their clinical application. Moreover, the NMM approach employed in the VEP process reduces source activity of thousands of vertices into a single node that corresponds to a VEP region. The VEP method may be improved by utilizing high-resolution imaging to increase the predictive power. Last, the VEP method does not account for the daily-to-monthly, irregular and regular dynamic patterns of epileptic seizures that exhibit circadian or cluster organization [10].

## Conclusions

Patient-specific virtual brain models may be a compelling option in clinical practice. The unique and untapped potential of virtual brain models remain to be exploited in the fields of neuroscience, medicine, and neurotechnology.

## Data Availability

Not applicable.

## Abbreviations

DWI: Diffusion-weighted imaging
EI: Epileptogenicity index
cEI: Connectivity epileptogenicity index
EZN: Epileptogenic zone network
NMM: Neural mass model
SEEG: Stereoelectroencephalography
VEP: Virtual epileptic patient
