# Correction: Emerging personalized virtual brain models: next-generation resection neurosurgery for drug-resistant epilepsy?

**DOI:** 10.1186/s42494-023-00141-4

**Published:** 2023-12-18

**Authors:** Qiao Wang, Guangyuan Jin, Tao Yu, Fabrice Bartolomei, Liankun Ren

**Affiliations:** 1 Department of Neurology, Xuanwu Hospital, Clinical Center for Epilepsy, Capital Medical University, Beijing, 100053 China; 2 National Center for Neurological Disorders, Beijing, 100070 China; 3 Department of Functional Neurosurgery, Beijing Institute of Functional Neurosurgery, Xuanwu Hospital, Clinical Center for Epilepsy, Capital Medical University, Beijing, 100053 China; 4 APHM, Epileptology and Clinical Neurophysiology Department, Timone Hospital, 13005 Marseille, France; 5 Aix-Marseille Université, Institut National de La Santé Et de La Recherche Médicale, Institut de Neurosciences Des Systèmes (INS) UMR1106, 13005 Marseille, France; 6 Chinese Institute for Brain Research, Beijing, 102206 China; 7 Department of Neurology, Xuanwu Hospital, Capital Medical University, NO.45 Changchun Street, Beijing, 100053 Xicheng District China

**Correction: Acta Epileptologica 5, 17 (2023)**

**https://doi.org/10.1186/s42494-023-00128-1**

Following publication of the original article [1], the authors reported an error in the image and legend of Fig. 1.

The original legend of Fig. 1 is:

Application of personalized virtual brain modeling in drug-resistant epilepsy: from bench to bedside

The revised image (with panel labels added) and legend of Fig. 1 is shown in the below content.

**Fig. 1:**
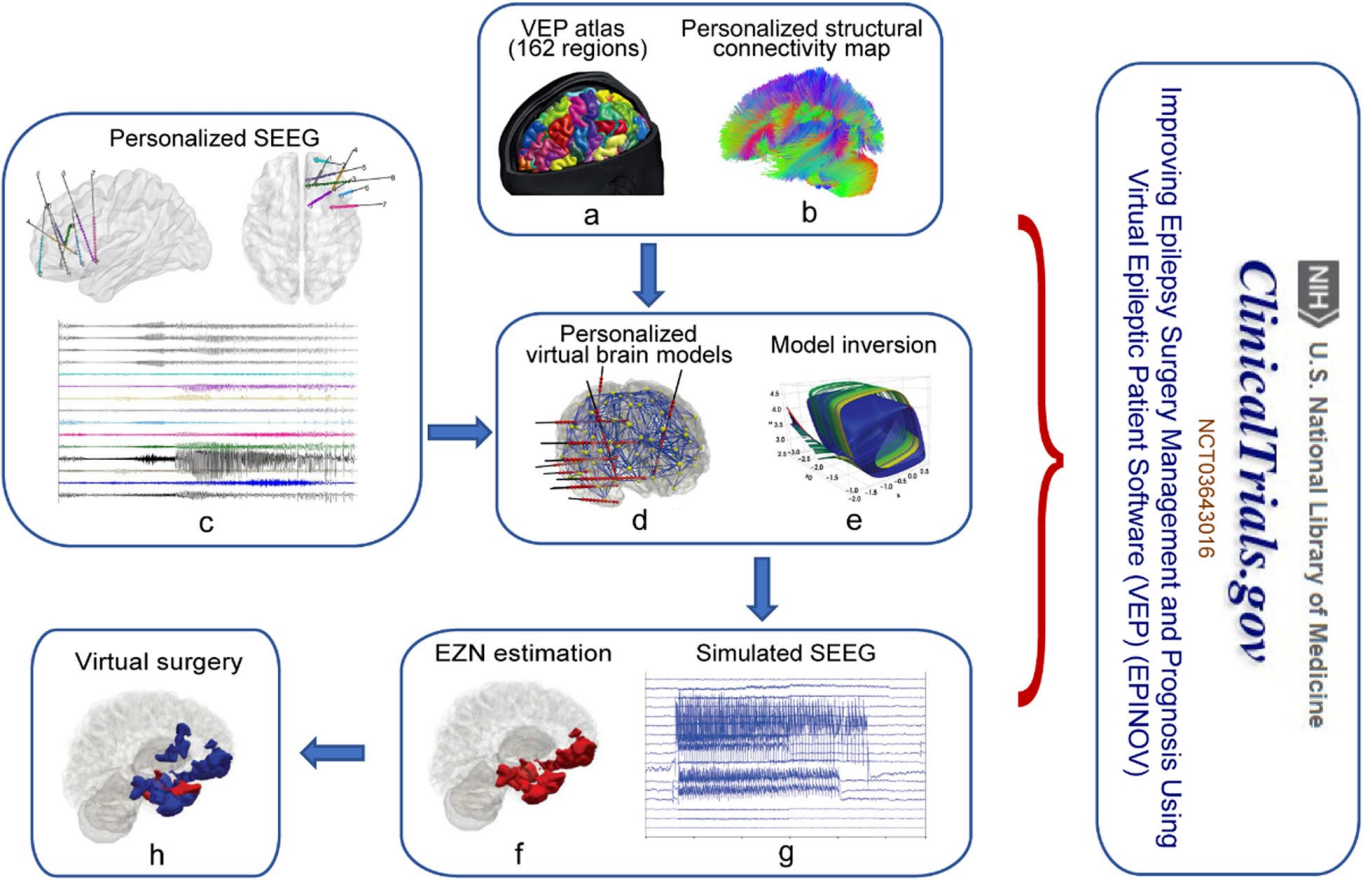
Application of personalized virtue brain modeling in drug-resistant epilepsy: from bench to bedside. First, a T1-weighted MRI is utilized to acquire brain anatomy and delineate distinct brain regions based on the Virtual Epileptic Patient atlas (Fig. 1a) as the nodes in the network model. The links between the nodes of the network are estimated based on patient-specific structural connectivity map (Fig. 1b) calculated from a diffusion-weighted imaging. Then, each node was assigned a neural mass model to simulate the average neuronal activity at that node. The Bayesian inference methods are used to estimate the patient-specific parameters of each NMM by fitting the simulated source activity (Fig. 1g) to the corresponding SEEG signals (Fig. 1c) with consideration of prior knowledge, a process called model inversion (Fig. 1e). Finally, a personalized brain model is constructed (Fig. 1d) and the output of the VEP workflow is the suggested epileptogenic zone networks (Fig. 1f), and the personalized model can be used to test different surgical strategies (Fig. 1h). Permission was granted by Viktor Jirsa et al. (©Elsevier [5]) to reuse this figure (a, d and g). Permission was granted by Huifang E. Wang et al. (©American Association for the Advancement of Science [4]) to reuse this figure (e, f and h)

The original article [1] has been updated.
